# Phosphoinositide 3-Kinase (PI3K) Subunit p110δ Is Essential for Trophoblast Cell Differentiation and Placental Development in Mouse

**DOI:** 10.1038/srep28201

**Published:** 2016-06-16

**Authors:** Xiwen Hu, Jiangchao Li, Qianqian Zhang, Lingyun Zheng, Guang Wang, Xiaohan Zhang, Jingli Zhang, Quliang Gu, Yuxiang Ye, Sun-Wei Guo, Xuesong Yang, Lijing Wang

**Affiliations:** 1 Vascular Biology Research Institute, Guangdong Pharmaceutical University, Guangzhou 510006, China; 2 School of Basic Courses, Guangdong Pharmaceutical University, Guangzhou, 510006, Guangdong, China; 3 Key Laboratory for Regenerative Medicine of the Ministry of Education, Division of Histology & Embryology, Medical College, Jinan University, Guangzhou 510632, China; 4 Shanghai OB/GYN Hospital, Fudan University, Shanghai 200011, China; 5 Shanghai Key Laboratory of Female Reproductive Endocrine-Related Diseases, Fudan University, Shanghai, China

## Abstract

Maternal PI3K p110δ has been implicated in smaller litter sizes in mice, but its underlying mechanism remains unclear. The placenta is an indispensable chimeric organ that supports mammalian embryonic development. Using a mouse model of genetic inactivation of PI3K p110δ (p110δ^D910A/D910A^), we show that fetuses carried by p110δ^D910A/D910A^ females were growth retarded and showed increased mortality in utero mainly during placentation. The placentas in p110δ^D910A/D910A^ females were anomalously anemic, exhibited thinner spongiotrophoblast layer and looser labyrinth zone, which indicate defective placental vasculogenesis. In addition, p110δ was detected in primary trophoblast giant cells (P-TGC) at early placentation. Maternal PI3K p110δ inactivation affected normal TGCs generation and expansion, impeded the branching of chorioallantoic placenta but enhanced the expression of matrix metalloproteinases (MMP-2, MMP-12). Poor vasculature support for the developing fetoplacental unit resulted in fetal death or gross growth retardation. These data, taken together, provide the first *in vivo* evidence that p110δ may play an important role in placental vascularization through manipulating trophoblast giant cell.

Extensive research has shown that most of the major roadblocks hindering embryonic development occur during major transitions in the development of the placenta^1^,^2^,^3^,^4^,^5^, a remarkable chimeric organ that enables mammalian growth development of embryo/fetus^6^. In rodents, mature placenta is morphologically and functionally divided into three major components, including the outside maternal deciduas, the middle junctional zone and the innermost labyrinth^7^,^8^. The junctional zone consists of the utmost primary trophoblast giant cells (GCs) and glycogen trophoblast that directly interacts with maternal decidual cells, and spongiotrophoblast (SpT) that forms a distinct cellular layer overlaying the labyrinth zone, which is the inner compartment proximal to the fetus and responsible for the maternal-fetal interchange of nutrients/wastes^6^,^9^,^10^ The fetal-derived cells interacting directly with maternal tissues are TGCs^8^,^11^,^12^. The placenta is derived from the outer single layer cells of blastocyst called trophectoderm. After successful implantation, cells in the trophectoderm stop dividing and differentiate to form primary TGCs of the parietal yolk sac (parietal TGCs, or P-TGCs), which line the implantation chamber and anatomize to form a diffuse network of blood sinuses that enable the early transport and exchange of nutrients and endocrine signals^13^. On the other side, the polar trophectoderm continues to proliferate and gives rise to trophoblast stem cells (TSCs) which subsequently form extraembryonic ectoderm and ultimately develops into the SpT layer and all types of trophoblasts in the labyrinth and a later wave of TGCs, called secondary TGCs, which are thought to derive from the differentiation of ectoplacental cone (EPC) precursors^14^. TGCs are endocrine in nature and characterized by their extremely large cytoplasm, mononuclear and polyploid that result from endoreduplication^15^. During later gestation, TGCs secrete a wide array of hormones and cytokines, including steroid hormones and prolactin-related cytokines, to target the maternal physiological systems (maternal endocrine and immune systems) for proper maternal adaptations to pregnancy and the fetal-maternal interface to ensure vasculature remodeling^12^,^13^,^16^,^17^. These complex activities are sensitive to disruption, as shown by the high incidence of early embryonic mortality and pregnancy failures well documented in humans, as well as numerous peri-implantation lethal mutations in mice^1^,^13^.

Trophoblast invasion is a tightly regulated process involving interaction between maternal decidual cells and fetal trophoblast cells. Decidual cells secrete the highest levels of matrix metalloproteinases (MMPs) and their invasive potential increases in the presence of TGCs^18^. In first-trimester human placenta, MMP-2 expression/activity is observed in extravillous trophoblasts^19^,^20^, and MMP-12 functions in cell adhesion, elastin degradation, and extracellular matrix remodelling. Harris *et al.* identified MMP-12 as one of the most highly expressed protease genes in extravillous trophoblasts, could degrade elastin during vascular remodelling in the placenta^21^. Perturbation in the fine balance in MMPs may result in vascular changes associated with complications of pregnancy such as preeclampsia (PE)^22^,^23^,^24^,^25^.

The phosphatidylinositol-3 kinase (PI3K) pathway regulates numerous aspects of cell function, including cells migration, growth, differentiation, proliferation, apoptosis, metabolism and intracellular trafficking and tumorigenesis. Class I PI3Ks, which are heterodimeric complexes comprising a p110 catalytic subunit (α, β, γ, or δ) and a p85 regulatory subunit, mediate the recruitment of Akt to phosphotyrosine-containing signalosomes and have been reported recently as a key pathway in trophoblast cell development^26^,^27^. Among class IA catalytic subunits, p110α and p110β are ubiquitously expressed in leukocytes^28^,^29^ and the deletion of either result in early embryonic lethality due to defects in angiogenesis^30^. Originally identified through p110δ role in immune responses^31^, p110δ has been demonstrated via gene targeting studies in mice to be essential in maintaining the function of immunocytes and disrupted PI3K p110δ signaling dysregulates maternal immune cells and increases fetal mortality in mice^32^,^33^,^34^,^35^. Our group reported recently that p110δ also plays an essential role in physiological vascular regulation through regulating AP-1/MMP-12 pathway in mice^36^. Quite serendipitously, we discovered the litter size is significantly reduced during breeding of p110δ mutant inactive mice (p110δ^D910A/D910A^) as compared with that of the wild-type (p110δ^WT^). Consequently, we suspected that p110δ may play a pivotal role in murine gestation.

In this study, we evaluated the reproductive capacity and examined related histology and morphology in mice p110δ^D910A/D910A^ (all genotypes mentioned in this paper were maternal types). We first demonstrated that p110δ is highly expressed in P-TGCs, which can facilitate initial maternal vascular connections, induce uterine decidualization, regulate cell differentiation and maternal physiology, produce angiogenic and hematopoietic hormones, and may be involved in establishing the parietal yolk sac before circulation into the mature placenta. These results demonstrate that p110δ is a crucial PI3K component that mediates TGCs function and thus placentation.

## Results

### Maternal PI3K p110δ Inactivation Reduces Litter Sizes inMice

**Figure 1:**
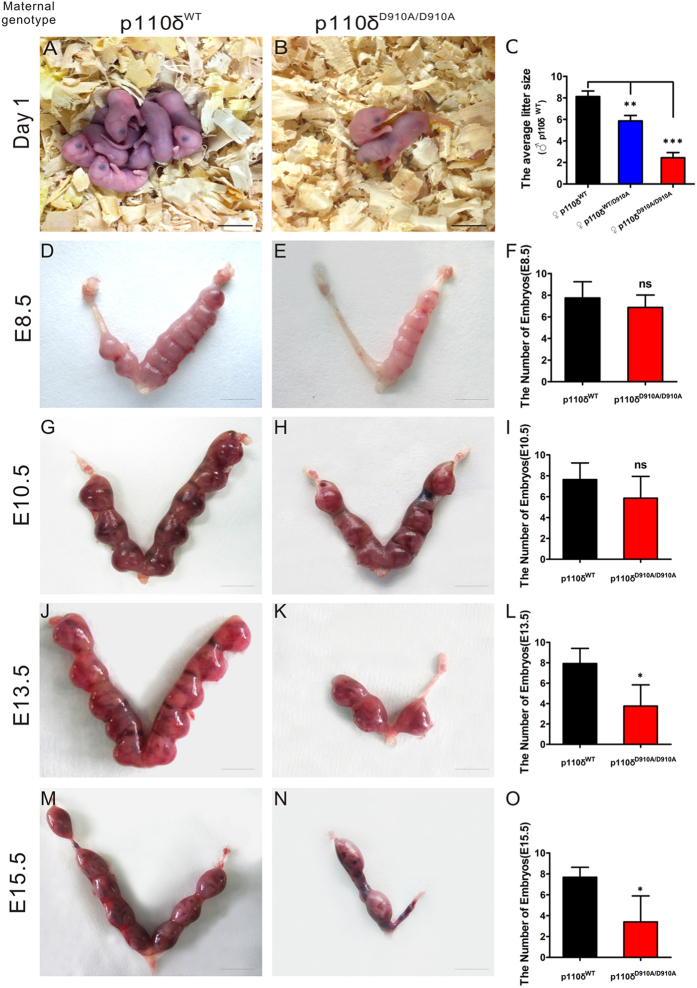
The Embryonic Miscarriage in p110δ Inactive Female Mice. Different p110δ genotype female mice were mated with p110δ^WT^ male mice, respectively. (**A**) A representative litter from p110δ^WT^ female mice. (**B**) A representative litter from p110δ^D910A/D910A^ female mice. (**C**) The comparison of average litter sizes among different mating (p110δ^WT^ group (n = 8), p110δ^WT/D910A^ group (n = 14) and p110δ^D910A/D910A^ group (n = 9)). (**D,E,G,H,J,K**) The different gestational stages of uterus in p110δ^WT^ (n≥4) and p110δ^D910A/D910A^ (n≥4) female mice. The statistical results of each testing stage (**F,I,L,O)**. The statistical data are expressed as the mean ± S.D. *P < 0.05, **P < 0.01, ***P < 0.001 and ns: P > 0.05. Scale bars: 8 mm (**A**,**B**), 4 mm (**D,E,G,H**) 5 mm (**J,K**) and 10 mm (**M,N**).

Previous reports have demonstrated that homozygous p110δ inactive mice (p110δ^D910A/D910A^) were indeed viable and fertile, without any gross anatomical or behavioral abnormalities, unlike p110α/β mutant mice^30^,^37^,^38^. However, we found, quite serendipitously, that the p110δ^D910A/D910A^ mice had significantly reduced litter size (see Fig. 1A–C and Supplementary Fig. S1A). To elucidate its causes, we mated p110δ^WT^ male mice with female mice with three different p110δ-genotypes: p110δ^WT^, p110δ^WT/D910A^ and p110δ^D910A/D910A^, respectively. The matings showed a normal Mendelian ratio at weaning without sex selection (see Supplementary Fig. S1C). Detailed genotyping studies showed that the reduction in litter size in p110δ inactive mice has a close relation with the maternal p110δ inactivation (see Supplementary Fig. S1b). As shown in Fig. 1C, the maternal p110δ inactivation showed reduced litter sizes as compared with the wild-type, and remarkably, the homozygous p110δ inactivation had about 4 folds of reduction in litter size as compared with their wild-type counterpart. Further observations of gestational uterus showed that embryo/fetus was lost in the early post-implantation period, and the reabsorbed aborted tissues were usually found from E10.5 till the birth (Fig. 1D–O). These results suggested that p110δ is essential for the normal growth and development of embryo/fetus.

### Maternal p110δ Inactivation Impedes Normal Placentation in Mice

**Figure 2:**
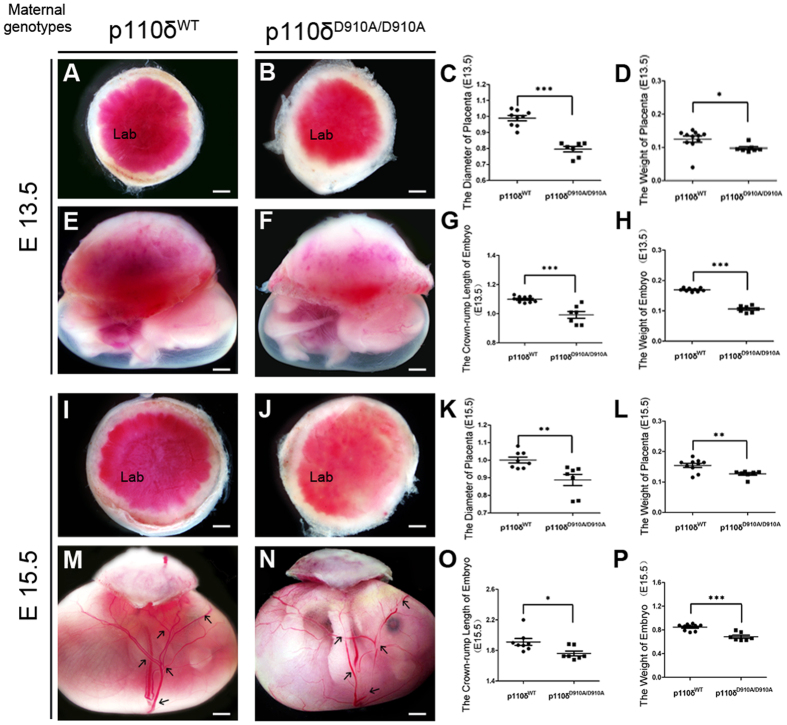
The Anomalous Placenta and Fetus in p110δ Inactive Female Mice. Morphological observation of placenta and fetus. The representative appearance of placentas from p110δ^WT^ and p110δ^D910A/D910A^ female mice at E13.5 (**A,B**) and E15.5 (**I,J**). The comparison of the diameters and weights of the placentas from p110δ^WT^ (n = 9), and p110δ^D910A/D910A^ (n = 7) female mice at E13.5 (**C,D**) and E15.5 (**K,L**). The representative appearance of the fetus from p110δ^WT^ and p110δ^D910A/D910A^ female mice at E13.5 (**E,F**) and E15.5 (**M,N**). The comparison of fetal crown-rump lengths and weights from p110δ^WT^ (n = 9) and p110δ^D910A/D910A^ (n = 7) female mice at E13.5 (**G,H**) and E15.5 (**O,P**). Lab, Labyrinth zone. The statistical data are expressed as the mean ± S.D. *P < 0.05, **P < 0.01 and ***P < 0.001. Scale bars: 1 mm (**A,B,E,F,I,J**), 500 μm (**M,N**).

Lethality in the early post-implantation period is indicative of defects in either implantation, placental or yolk sac^1^,^39^. The placenta is an indispensable chimeric organ that supports mammalian embryonic development. We then carefully examined the the gross anatomy of both placentas and the related fetus (Fig. 2. The genotypes mentioned referred to the maternal ones.). Anomalously anaemic appearance of the placenta was observed in the p110δ^D910A/D910A^ group. The weight and diameter of placentas were significantly reduced than that of p110δ^WT^ (Fig. 2A–D,I–L), at E15.5, and the placentas were noticeably much more anaemic or paler in labyrinth zone anatomically. In addition the vascular network in the fetal membrane appeared discontinuous and incomplete at E15.5 (Fig. 2M,N), which likely indicates angiogenic defects in placentas. Accordingly, neither deformity of the fetal appearance nor malformation of the heart and brain were observed apart from reduced weight and the crown-rump length (Fig. 2E–H,M–P).

**Figure 3:**
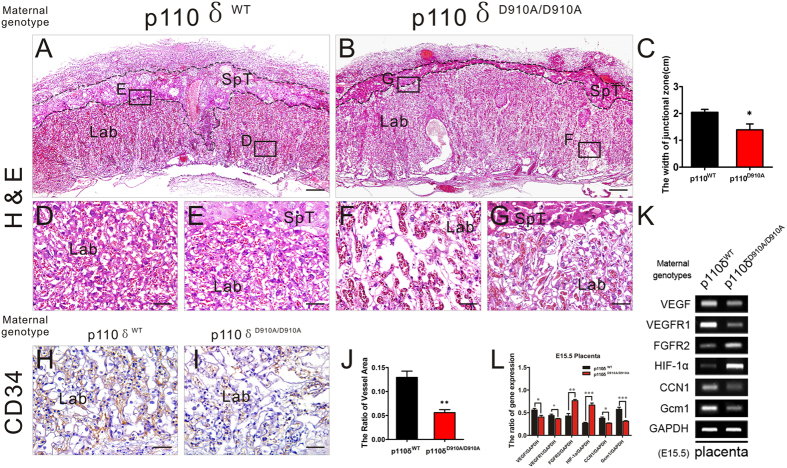
The Defects of Placental Vascularization in p110δ Inactive Female Mice. Histological appearance of the placenta. (**A,B**) The representative H&E stained transverse sections of placentas from p110δ^WT^ (**A**) and p110δ^D910A/D910A^ (**B**) female mice at E15.5. (**C**) The statistical result about the width of junctional zone in placentas from p110δ^WT^ (n = 7), and p110δ^D910A/D910A^ (n = 8) female mice at E15.5. (**D,G**) Higher magnification images of labyrinth zone from the sites that indicated by dotted squares in (**A,B**). (**H,I**) The immunohistochemical test against CD34 antibody on transverse sections of placentas. (**J**) The statistical result about the quantity of vessels in the labyrinth zone (**K**) The RT-PCR data showed the mRNA expression of placental VEGF, VEGFR1, FGFR2, HIF1-α, CCN1 and Gcm1 of E15.5 placenta in p110δ^WT^ and p110δ^D910A/D910A^ female mice. (**L**) The statistical result of RT-PCR. The statistical data are expressed as the mean ± S.D. *P < 0.05, **P < 0.01, ***P < 0.001 and ns: P > 0.05. Scale bars: 500 μm (**A,B**), 50 μm (**D–I**).

The findings suggest that the maternal p110δ inactivation have caused defects very likely in the placental vascularization. Histological analyses of midsagittal sections of E15.5 placentas showed that the organization of junctional zone (indicated by dotted line in Fig. 3A,B,D–G, Supplementary Fig. S2) including primary trophoblast giant cell (P-TGC) and spongiotrophoblast (SpT) layers from p110δ inactive female mice looked similar to that of control group apart from reduced thickness ratio. However, a conspicuously anomalous labyrinth layer, shownin Fig. 3A–C, was the disrupted blood sinuses network. And the layer is the place that supports the transport of nutrients, gases, ions, hormones as well as waste between the mother and fetus during gestation^40^,^41^. Unlike p110δ^WT^ group, the maternal blood sinuses and fetal capillaries in the labyrinth of p110δ^D910A/D910A^ group were apparently deficient (Fig. 3A–G). Morphometric and immunohistochemical analyses revealed that the density of the sinuses was reduced significantly in the p110δ^D910A/D910A^ group (Fig. 3H–J). To confirm, the expression of genes known to be essential for branching morphogenesis in the chorioallantoic placenta^13^,^42^,^43^,^44^ was evaluated by RT-PCR: vascular endothelial growth factor (VEGF), VEGFR1, fibroblast growth factor receptor 2 (FGFR2), hypoxia inducible factor (HIF-1α), CCN1 (Cyr61) and glial cell missing 1 (Gcm1) (Fig. 3K). Among these genes, the expression of VEGF, VEGFR1, CCN1 and Gcm1 was decreased significantly, while that of FGFR2 and HIF-1α was elevated significantly in p110δ^D910A/D910A^ group (Fig. 3L). These findings indicate that the maternal p110δ inactivation changed the placental structural proportion and disrupted the flexuous network of maternal and fetal vessels in the labyrinth.

### p110δ Expresses in Primary Trophoblast Giant Cells at Early Placentation

**Figure 4:**
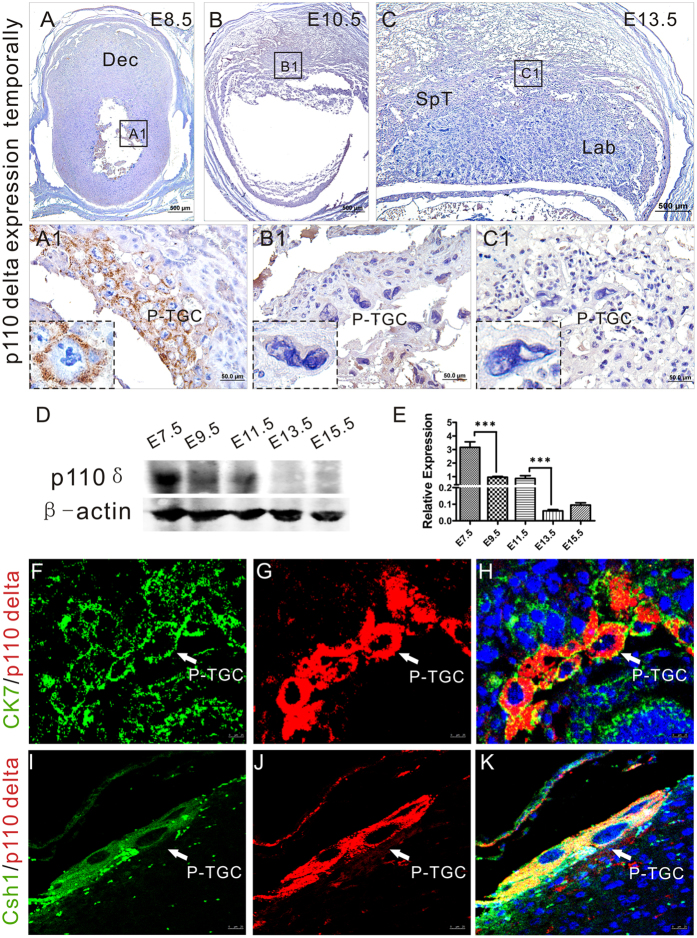
p110δ expresses in P-TGCs in early post-implantation period. p110δ expresses in P-TGCs. (**A–C**) Immunohistochemistry against p110δ antibody was performed on transverse sections of tissues from p110δ^WT^ female mice at E8.5 (**A**), E10.5 (**B**) and E13.5 (**C**). (**A1–C1**) Higher magnification images from the sites as indicated by the dotted box in (**A–C**). (**D**) Western Blot test at different stages in p110δ^WT^ placental biogenesis. (**E**) The statistical result of western blot data in (**D**). (**F–H**) Dual immunofluorescent test against p110δ and CK7 on gestational tissue at E8.5. (**I–K**) Dual immunofluorescent test against p110δ and Csh1 on gestational tissue at E8.5. Dec, decidua; P-TGC, primary trophoblast giant cell; Csh1, chorionic somatomammotropin hormone 1 (placental lactogen). The statistical data are expressed as the mean ± S.D. ***P < 0.001. Scale bars: 500 μm (**A–C**), 50 μm (**A1–C1**), 25 μm (**F–K**).

P-TGCs are critical for implantation and modulation in the post-implantation placentation^9^. As reported recently, the PI3K/Akt pathway is involved in trophoblast cell development^27^. In light of our findings presented above, we wondered whether PI3K-p110δ is functionally linked with P-TGC during placentation. We detected, by immunohistochemistry and Western blot analysis, the expression of p110δ in tissues from implantation to placentation in mice (Fig. 4A–E). As shown in Fig. 4A–C, the immunoreactivity against p110δ was strong and specific in cytoplasm of a set of cells, which were proved to be P-TGCs by dual immunofluorescent staining using antibodies against cytokeratin 7 (CK7, a marker for trophoblast cells) and chorionic somatomammotropin hormone 1 (Csh1, also called placental lactogen-1, a marker of early stage P-TGCs^45^), in post-implantation period (E8.5, Fig. 4A), and then gradually abated with gestation age (E10.5, E13.5, Fig. 4B,C). This can be seen more clearly in higher magnification that focused on a single P-TGC in the image (the blown-up portions of Fig. 4A1–C1). These results were also confirmed in Fig. 4D,E. According, the Akt signalingin P-TGCs was inactive in maternal p110δ inactivation group (see Supplementary Fig. S3). Taken togethor, the temporal expression of p110δ indicated that PI3K-p110δ may be involved in TGCs funtions (differentiation, invasion and secretion) in early placentation.

### Maternal p110δ Is Required for Normal TGCs Function

**Figure 5:**
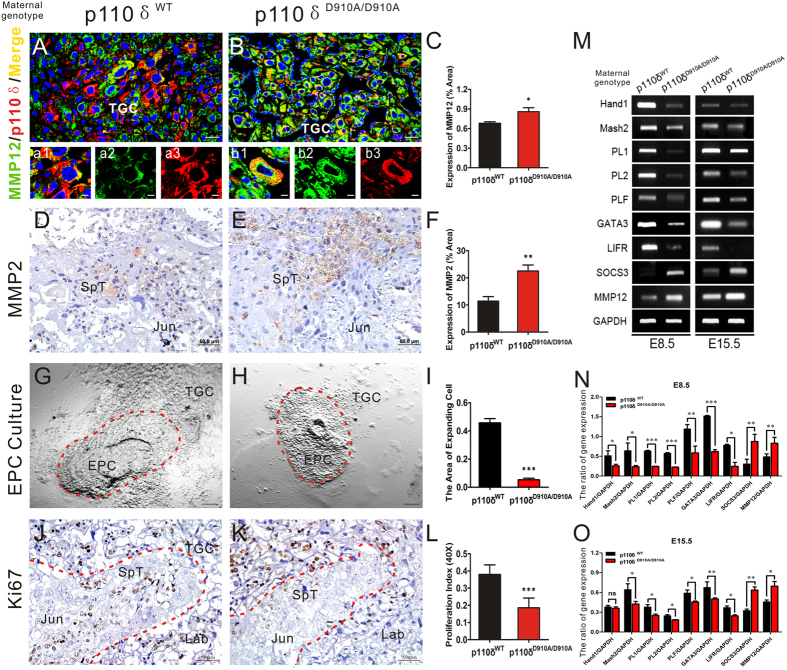
The capabilities of TGCs were restricted in inactive p110δ derived placentas. TGCs function was restricted in inactive p110δ. (**A,B**) Dual immunofluorescent test against p110δ and MMP12 on transverse sections of E8.5 tissue from p110δ^WT^ (n = 6) and p110δ^D910A/D910A^ (n = 6) female mice. (**C**) The statistical result of MMP12 expression in TGCs. (**D,E**) Immunohistochemistry test against MMP2 on transverse sections of E15.5 placenta from p110δ^WT^ (n = 6) and p110δ^D910A/D910A^ (n = 7) female mice. (**F**) The statistical result of MMP2 expression. (**G,H**) Bright-field images showed cultured EPC tissues from p110δ^WT^ (n = 3 mice) and p110δ^D910A/D910A^ (n = 4 mice) female mice, respectively. The TGCs that migrated out of original EPCs were indicated by red dotted lines in (**G,H**). (**I**) The statistical result of TGCs extension areas from the *in vitro* cultured EPC tissues. (**J,K**) The immunohistochemistry test against Ki67 on transverse sections of E15.5 placenta from p110δ^WT^ (n = 12) and p110δ^D910A/D910A^ (n = 11) female mice. The junctional zone, which was rich in SpT, was indicated with red dotted lines. (**L**) The statistical result of proliferation index. (**M**) RT-PCR results showed the mRNA expression of Hand1, Mash2, PL1, PL2, PLF, GATA3, LIFR, SOCS3 and MMP12 in deciduas at E8.5 and the placenta at E15.5 from p110δ^WT^ and p110δ^D910A/D910A^ female mice, respectively. (**N,O**) The quantitative analysis of genes mRNA expression. EPC, ectoplacental cone. The statistical data are expressed as the mean ± S.D. *P < 0.05, **P < 0.01, ***P < 0.001 and ns: P > 0.05. Scale bars: 25 μm (**A,B**), 50 μm (**D,E,J,K**), 200 μm (**G,H**).

Secreted by the endometrium, the MMP family plays crucial roles in regulating the decidualization and the invasive ability of early cytotrophoblasts, which is a prerequisite for a successful implantation and early post- implantation period. In our previous work, MMP-12 was found to be up-regulated in arteries of p110δ^D910A/D910A^ mice, leading to the degradation of extracellular matrix in the vessels^36^. Consequently, we evaluated the expressions of MMP-12 and MMP-2 in placenta of p110δ^WT^ and p110δ^D910A/D910A^ female mice, respectively (Fig. 5A–F)^20^,^21^,^46^,^47^. We found that MMP-12 and MMP2 mainly located in cytoplasm of trophoblast cells, and their expression levels were both elevated in p110δ^D910A/D910A^ group (Fig. 5C,F).

To further determine whether the defects in maternal p110δ inactive placental development were associated with P-TGCs, we cultured the ectoplacental cone (EPC), an excrescence formed by the proliferation of the polar trophectoderm of the blastocyst, from p110δ^WT^ (Fig. 5G) and p110δ^D910A/D910A^ female mice (Fig. 5H). The tissues were found to adhere to the culture dish after 24 hours incubation. And after 48 hours, the migration areas of newborn P-TGCs from EPCs were quantified to assess the differentiation and expansion capability of the TGCs. We found that the migration areas of the p110δ inactive P-TGCs were significantly reduced as compared with that of in p110δ^WT^ (Fig. 5I). Moreover, we performed immunohistochemistry staining of Ki67 on the transverse cross section of placenta and found the fewer Ki67-positive SpT cells were found in the junctional zone of p110δ^D910A/D910A^ group (Fig. 5J–L).

Several molecules were reported to be essential regulators in differentiation of SpTs, TGCs and labyrinthine syncytial trophoblast cells of the mature chorioallantoic placenta^43^,^48^,^49^,^50^. Therefore, we measured, by RT-PCR, the expression levels of these genes at gestation E8.5 and E15.5 (Fig. 5M). We found that the expressions of Hand1 (which induces TGC differentiation), Mash2 (which suppresses EPC differentiation), PL1, PL2, PLF (which is involved in TGC differentiation), GATA3 (which regulates the transcription of PL1), leukemia inhibitory factor receptor, LIFR (which is derivative of trophoblast) were all significantly reduced in p110δ^D910A/D910A^ group at E8.5 and E15.5, respectively. The expression of suppressor of cytokine signaling 3, SOCS3 (which regulates spongiotrophoblast function) and MMP-12 (which is involved in cells interaction and vascular formation) were significantly increased at both E8.5 and E15.5 (Fig. 5N,O). These results indicated that the maternal p110δ may well be required for normal TGCs differentiation, proliferation, secretion and invasion during post-implantation period.

## Discussion

Recent studies have highlighted the implantation and development of the placenta as major determinants of fetal growth and development^51^,^52^,^53^. The consensus is that most of the major roadblocks to in utero development occur during major transitions in the development of the placenta, which primarily affect the ability of the placenta to meet the increasing cardiovascular demands of the growing and developing fetus. The mammalian placenta connects the developing fetus to the maternal blood and nutrient supply through a complex vascular network. Molecular genetic studies in mice aimed at identifying potential regulators of these processes have been hampered by the lack of understanding of genetic functions in the placenta and the general nature of maternal–fetal interactions.

In this study, we confirmed that the litter size is decreased in p110δ^D910A/D910A^ female mice during breeding, which is consistent with the previous report^54^. The function of PI3K-P110δ has been investigated primarily in the immune system because it is mainly expressed on leukocytes, which is quite different from the ubiquitous expression pattern of p110α/β that was reported to affect embryonic angiogenesis. Our experimental data indicate that p110δ may also play a crucial role in reproduction and vascular physiology because it is specifically and in a distinct temporal and spatial fashion expressed on P-TGCs that line the implantation chamber, anastomosed to form a diffuse and extensive network of blood sinuses for the early transport and exchange of nutrients and endocrine signals, suggesting that its mutation has a deleterious effect on mouse placentation. Indeed, the abnormity occurs in mice during mid-gestation and exhibits defects in placental development. Compared with the p110δ^WT^ mice, these placentas are poorer in vascularization, smaller in size and lighter in weight in gross anatomy, and have a thinner or even absent junctional zone, sparser vascular network in the labyrinth zone and disrupted vascular connections in histomorphology.

Placental vascularization is formed in early pregnancy and supports fetal growth and development^55^,^56^,^57^,^58^. During a normal pregnancy, an adequate angiogenesis in maternal and placental tissues takes place and is always accompanied by a marked increase in uterine and umbilical blood flows^59^,^60^,^61^. This process is tightly regulated by numerous growth and angiogenic factors^62^,^63^,^64^,^65^. LIFR is expressed on trophoblast and mesodermal derivatives, and its deficiency results in the loss of giant cell differentiation and subsequent small labyrinth and vascular lesions^5^,^14^,^66^ SOCS3 deficient placentas exhibits reduced spongiotrophoblasts and suffers from embryonic lethality^66^. In addition, mutation in Gcm1 in mice causes a complete blockade of branching of the chorioallantoic interface and failure to fuse to form syncytiotrophoblast^67^,^68^, and CCN1 (Cyr61) null mice suffer embryonic lethality resulting from a failure in chorioallantoic fusion and placental vascular insufficiency and compromised vessel integrity^69^. The factors mentioned above were significantly decreased in placenta of p110δ mutant female mice as compared with that of the wild-type counterpart. Additionally, HIF-1α and FGFR2 expression was elevated in the p110δ^D910A/D910A^ female mice. As reported, fibroblast growth factor-4/FGF receptor-2 (FGF4/FGFR2) is also a factor implicated in the regulation of the trophoblast lineage^70^,^71^,^72^,^73^,^74^. Tissue oxygen levels and activation of the PI3K/Akt signaling pathway modulate HIF-1α protein levels^75^. Hypoxia is pivotal for the VEGF expression and the regulation of human trophoblast cells proliferation and differentiation via its transcriptional factor, HIF-1α^42^,^44^,^76^. In p110δ mutant female mice, defective branching of chorioallantoic placenta resulted in insufficient fresh blood supply, leading to an accumulation of undecomposed HIF-1α, a relatively high proliferation rate and poor differentiattion of trophoblast cells, but a reduction of the VEGF expression, which should be activated in hypoxia. It is still unclear and remains to be settled.

Through *in vitro* EPC tissue culture, we have demonstrated that the mutation of PI3K-p110δ significantly inhibited the extension of P-TGCs and several genes involved in TGC differentiation, such as Hand1, Mash2, PL1, PL2, PLF, and GATA3, are dysregulated in p110δ-mutant mice. Moreover, the numbers of TGCs and proliferation index of spongiotrophoblast cells in the junctional zone are significantly reduced in mutant mice as compared with that of the p110δ^WT^ mice. To examine the invasive and endocrine abilities of TGCs, we evaluated the expression of MMPs in placentas. The expression of MMP-12 elevated in TGCs, and MMP-2 also increased in the junctional zone. Trophoblast invasion is under strict control of tightly regulated and multiple interactions between maternal decidual cells and fetal trophoblast cells. The increased secretion of both MMP-12 and MMP-2 may be a potent inducement for TGCs invasion during placentation. The aberrant gene expression shown above is likely to account for defects of cell differentiation and vascular connections during mice placentation in p110δ mutants although further research is warranted to elucidate the precise mechanisms.

In humans, the polyploid cells, equivalent to TGCs in mice, are the so-called extravillous cytotrophoblast cells invading into the uterus^50^,^77^ and are associated with remodeling of the spiral arteries^78^. While some features of the gross anatomy and physiology of the mouse and human placenta are different, they share considerable cellular and molecular similarities. Many genes that are involved in TGC development and function, such as transcription factors, proteases and cell adhesion molecules, are conserved between rodents and humans^79^. Therefore, while direct observation on human placenta is clearly out of the question, the investigation of mice TGCs should give invaluable and much needed insights into human gestational disorders that are associated with extravillous cytotrophoblast cells such as preeclampsia and intrauterine growth restriction^80^.

**Figure 6:**
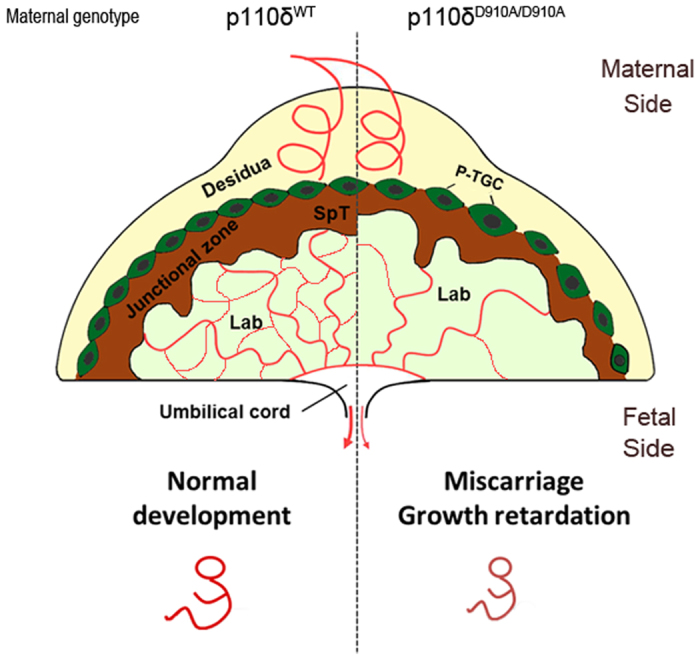
Illustration of a proposed model depicting the potential roles of p110δ in the placenta development.

In summary, using p110δ mutation transgenic mice, we have shown that p110δ may play crucial roles in the differentiation of trophoblast cells, vascularization and TGCs related MMPs expression (Fig. 6). Any serious defect in these aspects could lead to embryonic lethality due probably to insufficient blood supply from maternal-fetal connected placenta in mid-gestation. Despite the novel findings of the role of p110δ in TGCs in this study, the exact molecular mechanisms that regulate TGC differentiation, maternal-fetal vascular development and its relationship to feto-placental development would warrant more research.

## Materials and Methods

### Mice

C57BL/6 J (wide-type, p110δ^WT^) mice were purchased from Guangdong Medical Laboratory Animal Center. Mice with a kinase-dead mutation in the gene coding for PI3K p110δ (homozygous p110δ^D910A/D910A^ and heterozygous p110δ^WT/D910A^) in C57BL/6 J background have been previously described^37^. Mice were housed in the environmentally controlled specific pathogen-free animal rooms with lights on from 08:00–18:00 (10 h) and had access to food and water *ad libitum*. The morning that a vaginal copulation plug was detected was designated embryonic day (E) 0.5. Timed pregnancies were generated by cohabitation of female and male mice, and pregnant females were dissected at E6.5 through E15.5. The p110δ^D910A/D910A^ mice were bred and the conceptuses were PCR genotyped using genomic DNA prepared from tails or embryonic visceral yolk sac. Primers for genotyping were 5′-CTG TCA TCT CAC CTT GCT CC-3′; 5′-AGG GAA CCG CCG TAT GAC-3′; and 5′-AAT GCT TTC GTC CCA CGT CC-3′. All methods were carried out in accordance with the approved guidelines and all animal experimental protocols were approved by the animal experimental ethics committee of Guangdong Pharmaceutical University.

### Histology

Histological analysis was performed on at least three different placentas per stage of development (range of 3–10), obtained from more than three different mice. The pregnant mice were first euthanized and their uteri were removed by cutting at the cervix. For implacental samples, the uteri were fixed in 4% paraformaldehyde for 2–4 hours, then cut after every second or third implantation site and allowed to continue fixing overnight at 4 °C^17^. For placental one, the anti-mesometrial uterine muscle and membranes of each segment were cut open for photograph (Olympus SZX16, Japan), measurement (weight and length) and then fixed through immersion in 4% paraformaldehyde for 12 hours. They were cut into two pieces longitudinally and dehydrated in alcohol, embedded in paraffin blocks, and cut into 4 μm thick cross-sections. Serial sections were stained with hematoxylin and eosin (H&E) for general morphology. Additional sections were processed by immunohistochemistry to label cytokeratin 7 (Bioss, China; 1:150) of trophoblast cells, Chorionic somatomammotropin hormone 1 (Csh1/Placental lactogen, Bioss, China, 1:50) of trophoblast giant cells, CD34 (BOSTER, China; 1:100) of endothelial cells and p110δ (Santa Cruz, USA; 1:200), Akt (CST, USA; 1:100), p-Akt (CST, USA; 1:100), MMP2 (Sangon Biotech, China; 1:50), MMP9 (CST, USA; 1:100), MMP12 (Abcam, UK; 1:150), Ki67 (Genetech, China; 1:100). Formalin-fixed, paraffin-embedded sections were dewaxed, peroxidase-inactivated in 3% hydrogen peroxide for 30 min. Antigens was retrieved by heating in 0.01 M sodium citrate (pH = 6.0) for 10–20 mins. After three washes in PBS (pH = 7.2), tissue sections were blocked with 10% bovine serum albumin for 60 min in room temperature before incubating with the primary antibody at 4 °C overnight. After washed in PBS (pH = 7.2) again, horseradish peroxidase-conjugated secondary antibodies were used for detection. Immunohistochemistry staining was performed with diaminobenzidine (DAB, Thermo Fisher Scientific, USA) and counterstained with hematoxylin; Immunofluorescence was stained with DAPI. Isotope-matched IgG was used in place of the relevant primary antibody as a negative control. Sections from all mice were analyzed in parallel running, and images were captured using standardized procedures.

### Culture of EPC

EPC (ectoplacental cone, which is thought to give rise to secondary TGCs that can invade into the uterine deciduas at the late implantation stage^81^) from the mice on ~E7.5 of pregnancy was dissected out under sterile conditions and placed into wells coated with Matrigel (BD, USA) after washing by saline^8^. Cultures were kept in the incubator at 37 °C, in an atmosphere of 5% CO_2_. In the first 4–6 hours, the wells were kept turning over and stayed without medium. Then, the wells returned to normal condition and the medium Ham’s F-12 supplemented with 0.4% BSA (without covering the tissues) was added for continued culture for 48 h after the attachment (24 h) and outgrowth (48 h) were observed^82^.

### RNA isolation and RT-PCR

Tissue samples for RNA extraction were frozen in liquid nitrogen and stored at −80 °C. Total RNA was isolated from embryonic dissections using a Trizol kit (Invitrogen, USA) according to the manufacturer’s instructions. cDNA was synthesized to a final volume of 20 μl using Prime ScriptTM RT Reagent Kit (TaKaRa, Japan). Reactions were performed in a PCR machine (Biometra, Germany). The primer sequences were listed in Supplementary Fig. S4. The cDNAs were amplified for 35 cycles. One round of amplification was performed at 94 °C for 30 s, 30 s at 58 °C (±2 °C), and 30 s at 72 °C. The PCR products (25 μl) were resolved in 1.2% agarose gels (Biowest agarose, Spain) in 1× TAE buffer (0.04 M Trisacetate and 0.001 M EDTA). The resolved products were captured using a computer-assisted gel documentation system (Genetech, Shanghai,China)

### Western Blot analysis

The tissues were harvested and homogenized, and protein contents were measured using the BCA protein assay (Pierce). After blocking for 1 hour at room temperature in 5% milk solution, the membranes were probed overnight with primary antibody anti-p110δ (Santa Cruz). β-actin antibody was used as the internal control. After secondary antibodies were applied, final detection was carried out with an ECL Kit (Millipore). The data was analyzed by Image J software.

### Statistical analysis

Quantitative data are expressed as the mean value (mean ± standard deviation (SD)).The “n” represents the number of independent experiments on different mice. Data analyses and graphing were performed using the GraphPad Prism 5 software (GraphPad Software, CA, USA). The images of IHC and IF were analyzed with Image Pro Plus 6.0 software. The real-time PCR data were analyzed by an unpaired 2-tailed Student’s *t* test. *P* values of less than 0.05 and 0.01 were considered significant and very significant, respectively.

## Additional Information

**How to cite this article**: Hu, X. *et al.* Phosphoinositide 3-Kinase (PI3K) Subunit p110δ Is Essential for Trophoblast Cell Differentiation and Placental Development in Mouse. *Sci. Rep.* **6**, 28201; doi: 10.1038/srep28201 (2016).

## Supplementary Material

Supplementary Information
