# Author Correction: Phosphoinositide 3-Kinase (PI3K) Subunit p110δ Is Essential for Trophoblast Cell Differentiation and Placental Development in Mouse

**DOI:** 10.1038/s41598-020-63128-y

**Published:** 2020-04-20

**Authors:** Xiwen Hu, Jiangchao Li, Qianqian Zhang, Lingyun Zheng, Guang Wang, Xiaohan Zhang, Jingli Zhang, Quliang Gu, Yuxiang Ye, Sun-Wei Guo, Xuesong Yang, Lijing Wang

**Affiliations:** 1 Vascular Biology Research Institute, Guangdong Pharmaceutical University, Guangzhou, 510006 China; 2 School of Basic Courses, Guangdong Pharmaceutical University, Guangzhou, 510006 Guangdong China; 3 Key Laboratory for Regenerative Medicine of the Ministry of Education, Division of Histology & Embryology, Medical College, Jinan University, Guangzhou, 510632 China; 4 Shanghai OB/GYN Hospital, Fudan University, Shanghai, 200011 China; 5 Shanghai Key Laboratory of Female Reproductive Endocrine-Related Diseases, Fudan University, Shanghai, China

Correction to: *Scientific Reports* 10.1038/srep28201, published online 16 June 2016

This Article contains an error in Figure 5 M, as an incorrect image was used for Mash2 E15.5. The correct Figure 5 M appears below as Fig. 1.

**Figure 1:**
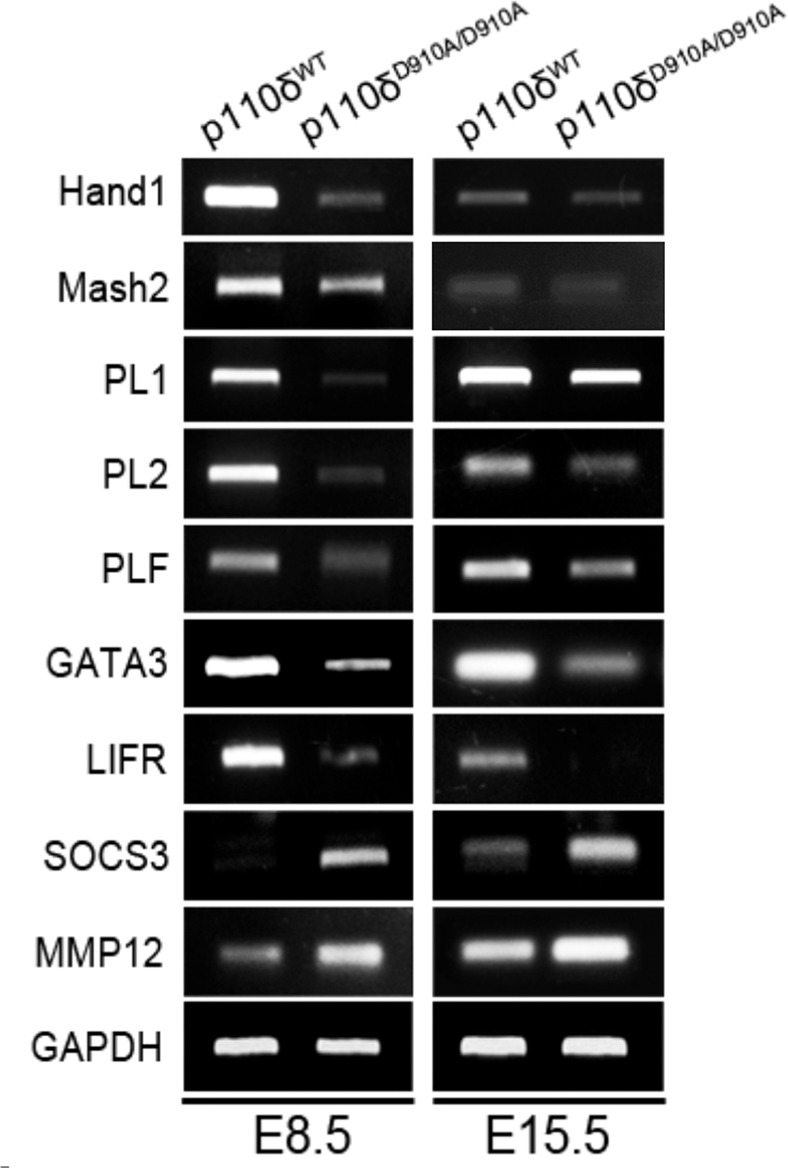
.

The original uncropped gel image for Mash2 E15.5 appears below as Fig. 2.

**Figure 2:**
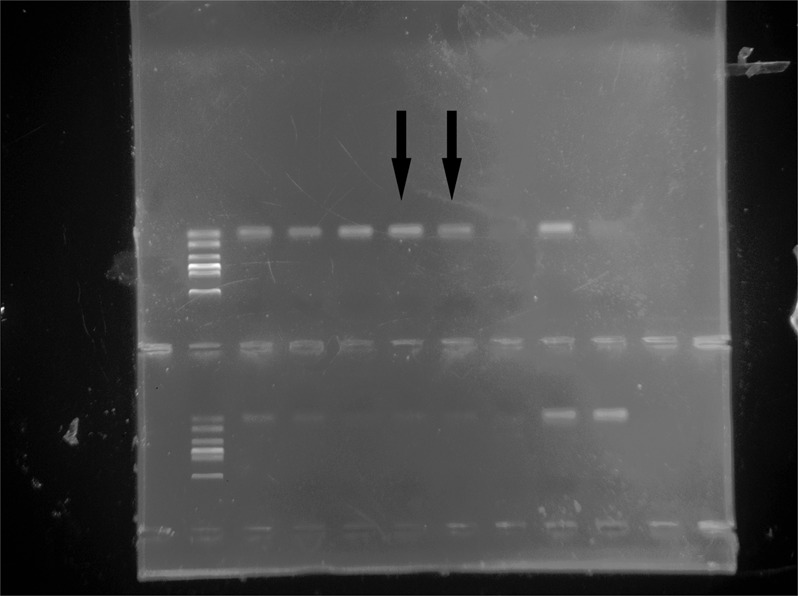
.

The conclusions of the Article are unaffected by these changes.

