# Supplementary material for: Phosphoinositide 3-Kinase (PI3K) Subunit p110δ Is Essential for Trophoblast Cell Differentiation and Placental Development in Mouse
Source: Sci Rep. 2016 Jun 16;6:28201. doi: 10.1038/srep28201 (PMC4910077; doi:10.1038/srep28201)
Supplement: Supplementary Information [file srep28201-s1.pdf]

Phosphoinositide 3-Kinase (PI3K) Subunit p110δ Is Essential for Trophoblast Cell Differentiation and Placental Development in Mouse

Xiwen Hu[1*], Jiangchao Li[1*], Qianqian Zhang[1], Lingyun Zheng[2], Guang Wang[3], Xiaohan Zhang[1], Jingli Zhang[1], Quliang Gu[1], Yuxiang Ye[1],Sun-Wei Guo[4], Xuesong Yang[3#] & Lijing Wang[1#]

[1]Vascular Biology Research Institute, Guangdong Pharmaceutical University, Guangzhou 510006, China;[2]School of Basic Courses, Guangdong Pharmaceutical University, Guangzhou, 510006, Guangdong, China; [3]Key Laboratory for Regenerative Medicine of the Ministry of Education, Division of Histology & Embryology, Medical College, Jinan University, Guangzhou 510632, China. [4]Shanghai OB/GYN Hospital, Fudan University, Shanghai 200011, China; and Shanghai Key Laboratory of Female Reproductive Endocrine-Related Diseases, Fudan University, Shanghai, China

[*] These two authors contributed equally to this work.

[#]Corresponding authors: LJ Wang, Ph.D., E-mail: wanglijing62@163.com or X Yang, Ph.D., E-mail: yang_xuesong@126.com

Conflict-of-interest disclosure: The authors declare no conflicting financial interests.

Supplementary Figure S1

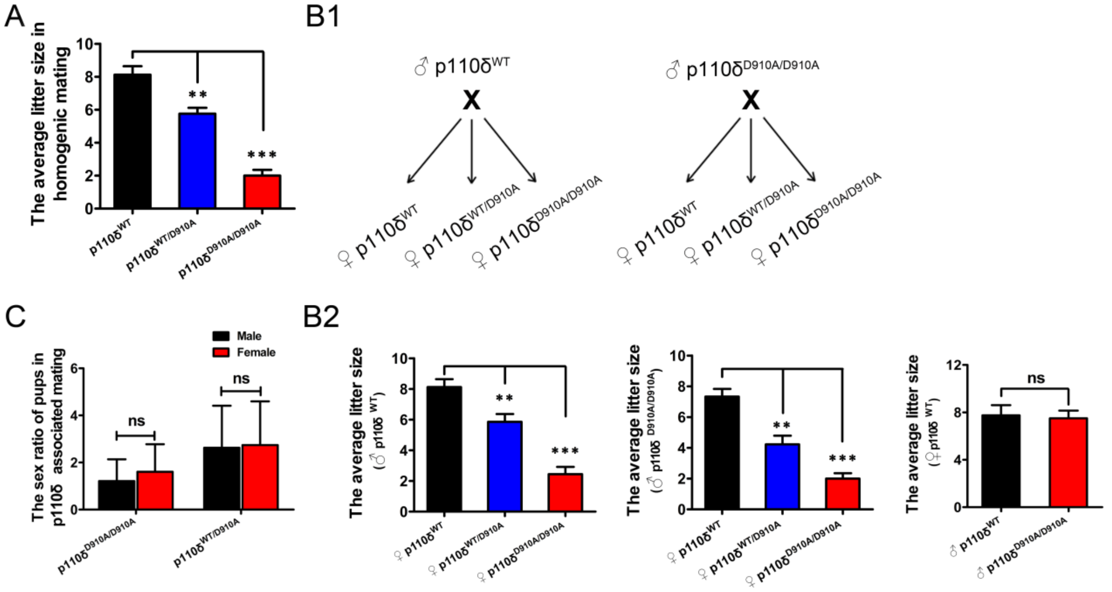

The statistical analyses of mice mating.

A. The statistical results of average litter size in three different homogenic mice mating. **B1-B2.** The detailed genotyping breeding studies in inactive p110δ mice. **C.** The sex ratio of pups in inactive p110δ mice mating.

Supplementary Figure S2

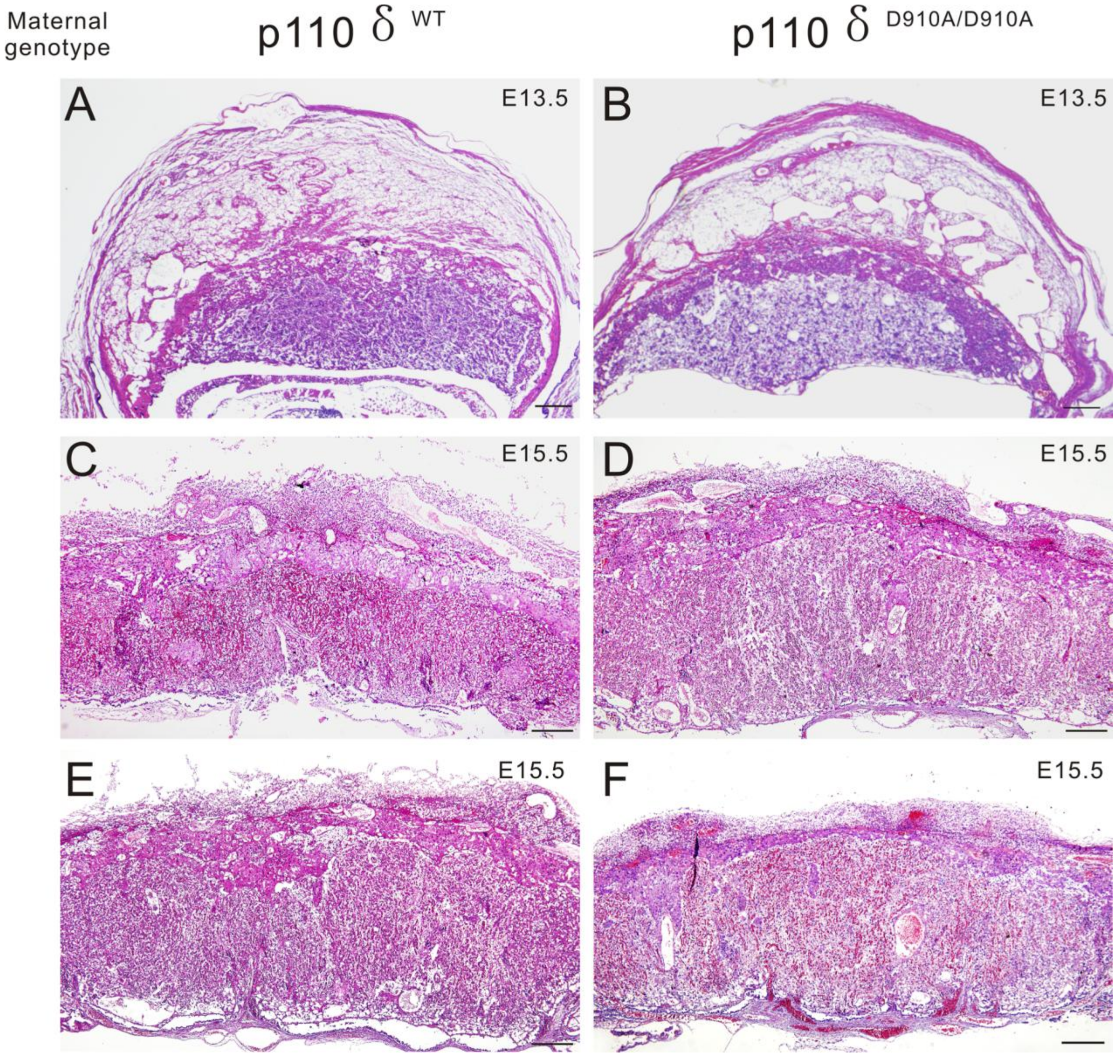

The additional histological observation of the placenta.

The H&E staining of placenta cross-sections at E13.5 (**A-B**) and at E15.5 (**C-F**) from p110δ^{WT} and p110δ$^{D910A/D910A}$ female mice, respectively. Scale bars: 500μm

Supplementary Figure S3

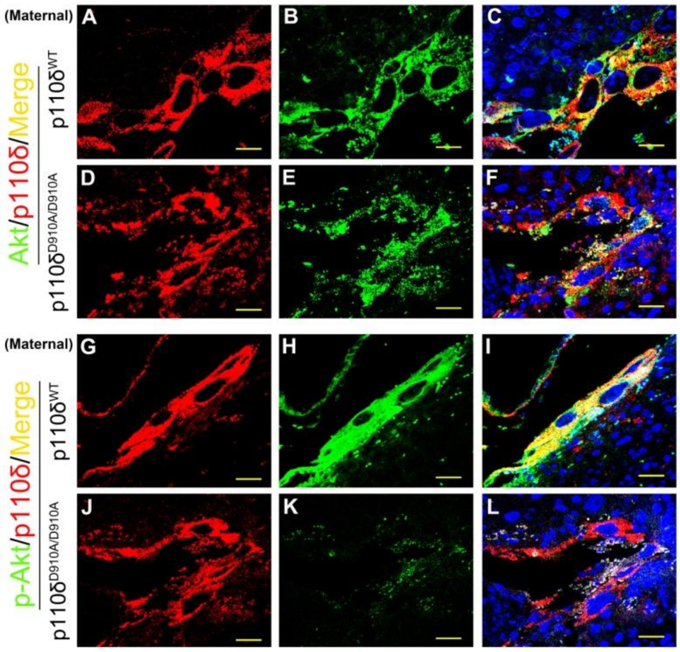

The activation of the PI3K/Akt signal in P-TGCs.

A-F. Dual immunofluorescent test against Akt and p110δ at E8.5 P-TGCs from p110δ^{WT} and p110δ$^{D910A/D910A}$ female mice. **G-L.** Dual immunofluorescent test against p-Akt and p110δ at E8.5 P-TGCs from p110δ^{WT} and p110δ$^{D910A/D910A}$ female mice. Scale bars: 25μm

Supplementary Figure S4

Name	Primer Sequences	
	F (5'–3')	R (5'–3')
Hand1	CATCGCCTACTTGATGGACGTG	CCCTTTAATCCTCTTCTCGCCG
Mash2	GTGCAAACGTCCACTTCCCACC	TGCTTTCCTCCGACGAGTAGGC
HIF-1α	GTCGGACAGCCTCACCAAACAGAGC	GTTAACTTGATCCAAAGCTCTGAG
Gcm 1	GATACTGAGCTGGGACATTAACG	CTGTCGTCCGAGCTGTAGATG
PLF	AGCCCCATGAGATGCAATACT	CGGACTGCGTTGATCTTTTTCTT
PL1	CCACTGAAGACCTGTATACTC	GGACTGCAGTTCTTCGAGTC
PL2	CACCAGACAACATCGGAAGAC	TGACAGCAGAGTATCAGGTACA
FGFR2	AGCGCCTGTGAGAGAGAAG	CCGAAACTGTTACCTGTCTCC
LIFR	AGCTCTGACCCTCCTGCAT	TGGGTGACAAGAATGGAACCT
SOCS3	TCACACTGAGCGTCGAGA	GTGGAGCATCATACTGGT
CCN1	CTCCAGAATCTACCAAAACGGG	CGTCCAGGGAGTCCTTAATGC
VEGFR1	CTCAGGGTCGAAGTTAAAAGTGC	TTGCCTGTTATCCCTCCCACA
VEGF	ATGACTTTCTGCTGTCTTGGGTG	CACCGCCTCGGCTTGTCACA
MMP12	TGAAGCGTGAGGATGTAGACT	TCAAGGATGGGGGTTTCACT
GATA3	GGAGGACTTCCCCAAGAGCA	CATGCTGGAAGGGTGGTGAG
GAPDH	TCCACCACCCTGTTGCTGTA	TCCACCACCCTGTTGCTGTA

The list of detail primer sequences used in RT-PCR experiment.
